# N6-methyladenosine methyltransferases: functions, regulation, and clinical potential

**DOI:** 10.1186/s13045-021-01129-8

**Published:** 2021-07-27

**Authors:** Wei Huang, Tian-Qi Chen, Ke Fang, Zhan-Cheng Zeng, Hua Ye, Yue-Qin Chen

**Affiliations:** 1 MOE Key Laboratory of Gene Function and Regulation, State Key Laboratory for Biocontrol, School of Life Sciences, Sun Yat-Sen University, Guangzhou, 510275 People’s Republic of China; 2 Department of Hepatobiliary, Sun Yat-Sen Memorial Hospital, Sun Yat-Sen University, Guangzhou, 510120 People’s Republic of China

**Keywords:** m6A, m6A methyltransferase, Cancer, Therapy resistance, Drug discovery

## Abstract

N6-methyladenosine (m6A) has emerged as an abundant modification throughout the transcriptome with widespread functions in protein-coding and noncoding RNAs. It affects the fates of modified RNAs, including their stability, splicing, and/or translation, and thus plays important roles in posttranscriptional regulation. To date, m6A methyltransferases have been reported to execute m6A deposition on distinct RNAs by their own or forming different complexes with additional partner proteins. In this review, we summarize the function of these m6A methyltransferases or complexes in regulating the key genes and pathways of cancer biology. We also highlight the progress in the use of m6A methyltransferases in mediating therapy resistance, including chemotherapy, targeted therapy, immunotherapy and radiotherapy. Finally, we discuss the current approaches and clinical potential of m6A methyltransferase-targeting strategies.

## Introduction

N6-methyladenosine (m6A) accounts for the most abundant mRNA internal modification [1] and is evident in long noncoding RNAs (lncRNAs) [2], microRNAs (miRNAs) [3], small nuclear RNAs (snRNAs) [4], small nucleolar RNAs (snoRNAs) [5] and ribosomal RNAs (rRNAs) [6, 7], thus covering almost the whole transcriptome. Although first detected in poly(A) RNA fractions in 1974 [8], interest in m6A was reestablished in 2012 when two groups described methylated RNA immunoprecipitation sequencing (MeRIP-Seq) [9, 10], a next-generation sequencing method used to map m6A throughout the transcriptome via a specific antibody. These mapping approaches revealed that m6A is preferentially found on a typical consensus sequence DRACH (D = G, A, or U; R = G or A; H = A, C, or U) and is highly dynamic, with levels that vary during development and in response to cellular stress, suggesting that m6A is likely to have functional roles that affect mRNA fate. The dynamic m6A level is known to be regulated by methyltransferases (also known as “writers”) and demethylases (also known as “erasers”). The m6A writers comprise a complex with METTL3, METTL14 and WTAP, as well as additional partner proteins to add m6A on mRNAs and other methyltransferase that catalyze the m6A modification on distinct RNAs, including the METTL5-TRMT112 complex, METTL16 and ZCCHC4, respectively [1, 11]. The erasers, including FTO [12] and ALKBH5 [13], remove the m6A modification from RNA. In addition, specific RNA-binding proteins that recognize m6A and affect the fate of RNAs are grouped as m6A readers, such as the YTH domain family of proteins [14] and IGF2BP proteins [15]. Currently, m6A has emerged as a widespread regulatory mechanism that controls gene expression [16].

The initiation and development of cancers usually result in disordered genomic and epigenetic regulation, which frequently leads to abnormal expression of a group of core genes associated with sustained proliferation, disrupted apoptosis, aberrant stemness and therapy resistance [17]. Increasing evidence has suggested that m6A methyltransferases drive a number of cancers due to their ability to determine RNA fate [16]. Interestingly, in various cancers, many m6A sites have been mapped to genes regulating the hallmarks of cancer, while the highly stable transcripts encoding “housekeeping” genes, such as ribosomal proteins, were found to be de-enriched m6A [18]. Furthermore, ablation of m6A methyltransferases has been shown to influence tumor progression [19]. These observations indicate that catalyzing the m6A modification of key genes involved in cancer biology might be crucial to their functions. Moreover, the m6A machinery, particularly methyltransferases, has been shown to contribute to therapy resistance, highlighting the potential for targeting RNA epigenetic mechanisms for the treatment of cancer.

In this review, we summarize recent progress in understanding the function and regulation of different m6A methyltransferase complexes, with a main focus on the METTL3-METTL14-WTAP complex, in the core signaling pathways of cancers and their roles in therapeutic resistance. We also highlight advances in m6A methyltransferase-targeting strategies and their clinical potential in cancer treatment.

## RNA m6A methyltransferases

The first writer was found prior to the recent resurgence in interest in m6A, when Bokar et al. cloned METTL3 in 1994 [20, 21]. To date, four methyltransferases have been found to be encoded in the mammalian genome and are known to modify distinct RNAs with m6A.

**Fig. 1:**
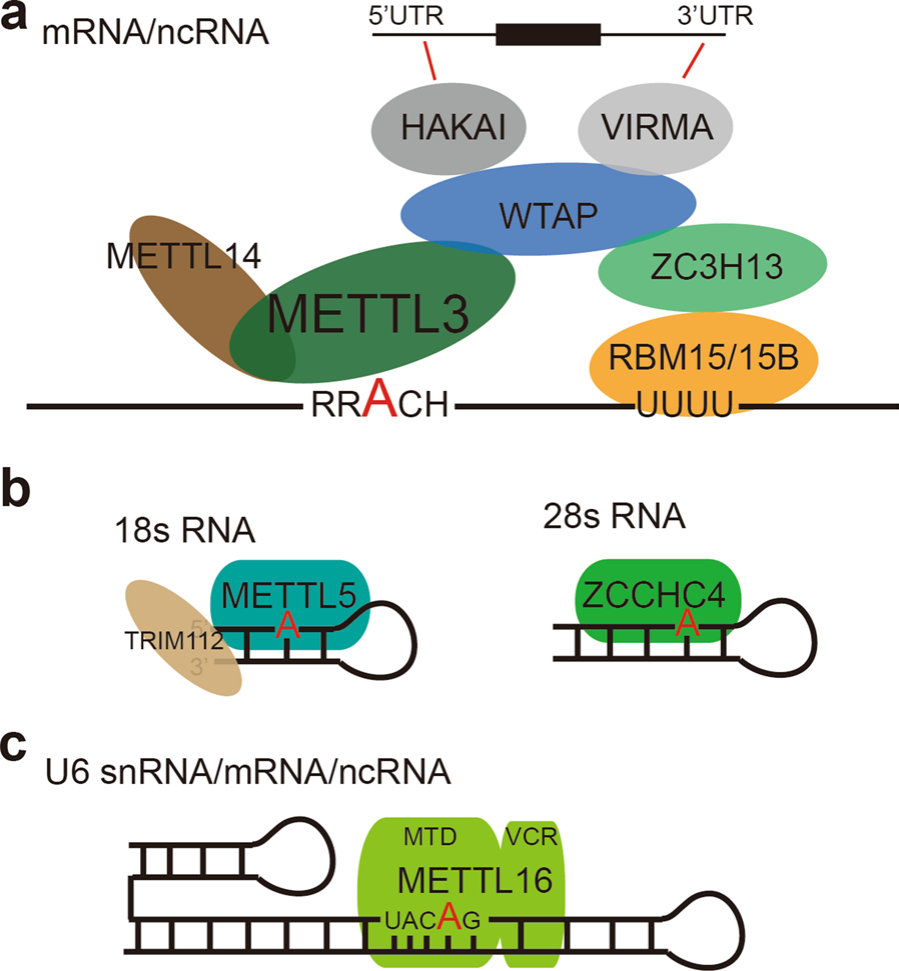
Schematic illustration of the reported m6A methyltransferase complex (MTC) of human RNAs. **a** Most of the mRNAs are methylated by the METTL3-METTL14 complex. This complex is composed of other adaptor proteins, including WTAP, VIRMA, RBM15/15B, ZC3H13 and HAKAI. These adaptors may help in determining the specific sites of m6A methylation. For example, VIRMA mediates preferential m6A mRNA methylation in the 3'UTR, while HAKAI affects m6A modification distributed in the 5’UTR. **b** m6A in 28S and 18S rRNA is the result of ZCCHC4 and METTL5-TRMT112 complex activity, respectively. **c** METTL16 mediates m6A modification in U6 snRNA and a small proportion of other mRNAs through specific structural recognition

The m6A methyltransferase complex (MTC) is composed of METTL3, METTL14, WTAP, VIRMA, RBM15/15B, ZC3H13 and HAKAI (Fig. 1a) and primarily modifies mRNAs and other RNA polymerase II-derived transcripts with m6A. METTL3 is the only catalytic subunit of MTC but is inactive without METTL14, which is catalytically inactive but essential in substrate recognition [22, 23]. Recent studies have indicated that other individual proteins in the MTC may have specific functions. For example, ZC3H13 is important for the nuclear location of the MTC [24], and WTAP anchors the complex to chromatin [25]. VIRMA mediates preferential m6A mRNA methylation in the 3'UTR and near the stop codon [26], while HAKAI affects m6A modification distributed in the 5’UTR and around the start codon [27]. RBM15 and RBM15B bind U-rich RNA consensus motifs [28] and thus may facilitate the recruitment of the MTC to specific sites in mRNA. However, why the m6A modification depends on such a large complex remains unknown, and the exact roles of each component have yet to be determined.

Although recent attention has focused on m6A in mRNA as mediated by the MTC, as we describe above, the vast majority of m6A in total cellular RNA is located on the much more abundant rRNAs. m6A modifications at position 1832 in human 18S rRNA and position 4220 in human 28S rRNA are catalyzed by the methyltransferases METTL5-TRMT112 complex [29] and ZCCHC4 [30], respectively (Fig. 1b, c). Among these enzymes, the METTL5 methyltransferase necessarily forms a heterodimer with TRMT112 to obtain metabolic stability. Importantly, these two m6A modification sites on rRNA are functionally important for mRNA translation. In addition to rRNA modifications, m6A in snRNAs is catalyzed by METTL16 [4], which can also mediate the m6A modification of U6-like sequences in MAT2A mRNA, which encodes the enzyme critical for S-adenosylmethionine (SAM) biogenesis, and in a small fraction of other mRNAs and noncoding RNAs [31].

The diverse m6A methyltransferases encoded in the genome confirm that m6A is an important posttranscriptional modification that is tightly regulated in organisms and requires multiple methyltransferases to determine RNA species-specific or site-specific methylation. Consistent with this idea, the dysregulation of m6A methyltransferases is highly associated with cancer initiation and progression.

## The functional and regulatory mechanisms of m6A methyltransferases in cancer biology

With the power to affect the function of the specific target RNA or a specific RNA area by the addition of methyl groups, m6A methyltransferases influence target RNA fate by regulating RNA stability, translation efficiency, splicing, and nuclear export [1]. In addition, m6A methyltransferases can separate from the MTC to regulate cancer progression independently. In this section, we discuss both the m6A-dependent and m6A-independent functions of m6A methyltransferases in regulating cancer biology.

## The m6A-dependent functions of m6A methyltransferases in key cancer pathway regulation

**Fig. 2:**
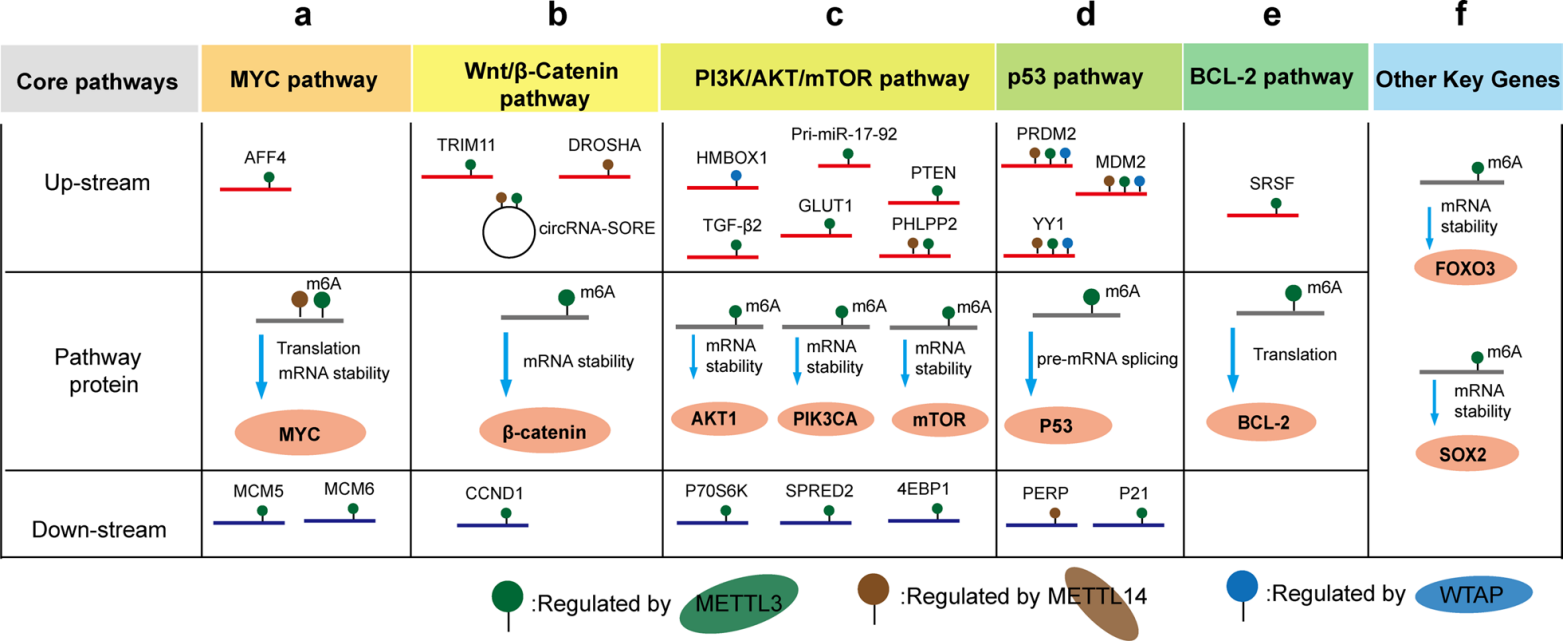
m6A methyltransferase regulation of the core pathways in cancer. The key cancer pathways, including the MYC pathway (**a**), Wnt/β-Catenin pathway (**b**), PI3K/AKT/mTOR pathway (**c**), p53 (**d**), BCL-2 (**e**), and other key genes (**f**), are regulated by the m6A methyltransferases METTL3/METTL14/WTAP. The schematic illustration shows the core genes in the related pathway that target different m6A methyltransferases, as well as the modulated upstream and downstream genes in these pathways

Within the past few years, extensive studies have suggested the pivotal role of m6A methyltransferases in cancer [16], and the crucial roles of m6A methyltransferases in regulating several key pathways have been reported, including MYC, Wnt/β-catenin, PI3K/AKT/mTOR, p53, BCL-2, and FOXO-SOX, which drive the cancer phenotype. Here, we summarize how m6A methyltransferases regulate core genes and pathways in cancer and discuss the underlying mechanism of their roles in tumorigenesis. The core pathway genes and upstream and downstream m6A-related regulatory genes are summarized in Fig. 2.

## MYC pathway

The MYC pathway is one of the most critical contributors to the genesis of many cancers, as it plays a central role in cancer initiation and progression by reprogramming a number of cellular processes [32]. MYC mRNA is universally reported to be regulated by m6A methyltransferases. In acute myeloid leukemia (AML), both METTL3 and METTL14 can directly target MYC to enhance its translation efficacy and inhibit differentiation and increase proliferation [33, 34]. In solid tumors, METTL3-mediated m6A modification of MYC mRNA regulates the stability of the corresponding RNA in urothelial carcinoma [35], prostate carcinoma (PC) [36] and oral squamous cell carcinoma (OSCC) [37], thereby enhancing tumor development. These studies clearly demonstrated that MYC is extensively regulated by m6A methyltransferases in a wide range of cancers and that targeting m6A methyltransferases can severely impair MYC-driven cancer phenotypes.

The upstream and downstream genes of MYC are extensively involved in cell growth and proliferation, as well as tumorigenesis. In bladder cancer, METTL3 mediates the m6A modification of the *AFF4* gene, which binds to the promoter of MYC and elevates MYC expression to promote cancer progression [35]. In gastric cancer, aberrant METTL3 expression is associated with poor prognosis of patients because of the m6A regulation of several key components (e.g., MCM5 and MCM6) in the MYC pathway [38]. The aforementioned observations, in which m6A methyltransferases are extensively involved in the MYC pathway and even the MYC gene itself (Fig. 2a), indicate that the targeting of m6A methyltransferases is an alternative approach to inhibiting MYC. Further studies are still needed to determine whether other important genes involved in the MYC pathway are regulated by m6A methyltransferases.

## Wnt/β-Catenin pathway

Components of the Wnt/β-catenin pathway are frequently mutated and/or overactivated in solid malignancies and promote tumor development [39]. In clear cell renal cell carcinoma (ccRCC), METTL3 and METTL14 constitute a risk signature for use in prognostics, because they are significantly enriched in cancer-related pathways, including the Wnt/β-catenin signaling pathway [40]. In hepatoblastoma (HB), METTL3-mediated m6A was reported to directly target β-catenin mRNA [41], leading to tumor growth in vitro and in vivo (Fig. 2b).

In addition, m6A methyltransferases affect upstream genes or cofunctional factors participating in the regulation of the Wnt/β-catenin pathway. In hepatocellular carcinoma (HCC), silencing METTL3 and METTL14 has been shown to reduce the level of circRNA-SORE, which acts as a miRNA sponge by recruiting miR-103a-2-5p and miR-660-3p to competitively activate the Wnt/β-catenin pathway, thereby inducing sorafenib resistance [42]. In nasopharyngeal carcinoma (NPC), METTL3 has been shown to induce high m6A enrichment in TRIM11, a tripartite motif-containing protein family member that positively modulates β-catenin signaling, to promote cisplatin resistance [43]. In breast cancer, RNase III DROSHA was reported to interact with β-Catenin to transactive STC1 in an RNA cleavage-independent manner, contributing to cancer stem cell properties. Interestingly, METTL14 can enhance DROSHA mRNA stability by catalyzing the m6A modification, indicating that targeting the METTL14-DROSHA-β-Catenin axis might impair breast cancer progression [44]. In addition, METTL3/WTAP and IGF2BP3 elevated the expression of β-catenin downstream gene, CCND1, via writing and reading the m6A modification on it to promote colon cancer progression [45] (Fig. 2b).

As the Wnt/β-Catenin pathway is a complex signaling cascade [46], the identification of novel genes in the Wnt/β-Catenin pathway, such as METTL3 and METTL14, might provide additional therapeutic opportunities in cancer treatment.

## The PI3K/AKT/mTOR pathway

The PI3K/AKT/mTOR pathway is a key signal transduction pathway activated by mitogen signals to induce cell cycle progression, cell proliferation, metabolism, and motility [47]. Genes in this pathway have been found to be commonly activated in cancer. In gastric cancer cell lines, mTOR, AKT1 and PIK3CA have been found to be hypermethylated by m6A methyltransferases [48]. Inhibiting the activity of methyltransferases (METTL3-METTL14 complex) by S-adenosylhomocysteine (SAH) or by METTL3 siRNA reduced the mRNA stability of AKT, PIK3CA and PTEN, consistent with the loss of m6A (Fig. 2c).

m6A methyltransferases can also activate the PI3K/AKT/mTOR pathway by targeting its upstream factors (Fig. 2c). METTL3 has been shown to induce GLUT1 translation to activate the m6A-GLUT1-mTORC1 axis and promote colorectal cancer (CRC) progression [49]. In osteosarcoma, WTAP mediates m6A modification at the 3’UTR of HMBOX1 mRNA, which downregulates its expression. The reduction in HMBOX1 leads to suppression of the PI3K/AKT pathway and inhibited tumor growth and metastasis in vivo and in vitro [50]. In addition, the negative regulator of PI3K/AKT, PTEN, has been reported to be a direct target of METTL3 in HCC [51] and chronic myelocytic leukemia (CML) [52]. Specifically, METTL3 regulates PTEN mRNA instability to reduce PTEN protein expression and cause continuous activation of the PI3K/AKT pathway in chronic myelocytic leukemia cells [52]. Sun et al. showed that METTL3 accelerates the maturation of pri-miR-17-92 and thus influences PTEN regulation of gastric cancer progression [53]. Other negative regulators of AKT pathways, including PHLPP2 [54], and TGF-β2 [55], have been reported to be direct targets of METTL3.

NF-kB is a major downstream molecule in the AKT pathway, and its activation is known to be involved in the immune response and inflammation. However, growing evidence also supports a role for NF-kB in oncogenesis [56]. Yin et al. revealed that loss of METTL3 impairs the YTHDF1-mediated translation of SPRED2, which enhances the activation of NF-kB and STAT3, leading to increased tumor growth and metastasis [57]. Other downstream genes of PI3K/AKT/mTOR can be regulated by m6A methyltransferases. METTL3 has been shown to elevate the mRNA levels of P70S6K and 4EBP1 to promote retinoblastoma (RB) progression [58].

The PI3K/AKT/mTOR pathway is considered a significant factor in the multidrug resistance (MDR) in a variety of cancers [59]. The strong connection between m6A methyltransferases and the PI3K/AKT/mTOR pathway indicates that m6A methyltransferases might serve as a hub to regulate MDR.

## The p53 pathway

The multifunctional molecule p53 acts as a potent barrier to cancer. Tumor-associated mutations in p53, which cause dramatic defects in p53 function, are hallmarks of most human cancers [60]. In 2012, a study showed that silencing m6A methyltransferases significantly affects gene expression, resulting in modulation of the p53 signaling pathway [10]. Another work illustrated the cooperative function of alterations in m6A and p53 and/or its regulator/downstream targets in the pathogenesis and maintenance of AML [61]. These studies clearly indicated that m6A participates in the p53 pathway. Indeed, an in-depth study showed that METTL3 catalyzes the m6A site at the point-mutated codon 273 (G > A) of p53 pre-mRNA to promote preferential pre-mRNA splicing [62]. As a result, the resultant p53 R273H mutant protein leads to acquired multidrug resistance in colon cancer cells, suggesting that p53 mRNA is a direct target of METTL3 (Fig. 2d).

In addition, the expression levels of METTL3, METTL14, WTAP, and KIAA1429 have been shown to be increased in arsenite-treated HaCaT cells, thereby elevating PRDM2, MDM2 and YY1 mRNA levels, resulting in p53 inactivation and arsenic carcinogenesis [63]. On the other hand, p21, also known as cyclin-dependent kinase inhibitor 1 (CDKN1A), is a direct target of p53 and mediates G1 growth arrest [64]. METTL3 can promote breast cancer cell proliferation by regulating p21 expression in an m6A-dependent manner [65]. Recent studies have suggested that the upregulation of METTL14 leads to a decrease in PERP, a p53 target gene involved in DNA damage-induced apoptosis, via m6A modification [66], thereby promoting the growth and metastasis of pancreatic cancer (Fig. 2d).

The current drugs that target the p53 pathway are aimed at inhibiting the protein–protein interaction between p53 and the E3 ubiquitin protein ligase MDM2 [67]. The observation that m6A methyltransferases extensively modulate the p53 pathway suggests an alternative enzyme-based strategy to drug invention. In particular, in addition to inducing the apoptotic death of cancer cells, the p53 pathway plays a role in preventing the development of cancer at the earliest point of induction. This surveillance function of p53 involves a distinct group of p53-induced genes that regulate DNA repair and metabolism [68]. It will be interesting to investigate whether m6A methyltransferase is involved in the p53 pathway related to cancer prevention and the potential of m6A methyltransferase targeting in chemoprevention.

## BCL-2 family proteins

Cancer development and progression are facilitated by enhanced cell survival signaling. BCL-2 family proteins, the key mediators of the apoptotic response, integrate stress and survival signaling pathways to exert their antiapoptotic function in cancer cells [69]. BCL-2 family expression has been reported to be associated with m6A-mediated RNA fate decisions (Fig. 2e). In breast cancer, METTL3 directly targets BCL-2 by increasing the extent of the m6A modification of BCL-2 to increase its RNA and protein expression levels, thereby regulating the proliferation and apoptosis of breast cancer [70]. In glioblastoma (GBM), silencing METTL3 or overexpressing dominant-negative mutant METTL3 has been shown to suppress the growth and self-renewal of glioma stem cells (GSCs). Mechanistically, METTL3 deficiency decreases m6A modification and SRSF mRNA levels, which leads to the downregulation of BCL-X isoform expression [71]. In addition to the abovementioned prosurvival members BCL-2 and BCL-X, the BCL-2 family of proteins contains proapoptotic proteins (e.g., BH3-only proteins, including BIM, PUMA, and BAD) to balance the fate decision between cell life and death [72]. How m6A methyltransferases are involved in BCL-2 family protein regulation is only beginning to be understood.

## Other key genes

m6A methyltransferases can also modulate other key genes in cancer biology. Forkhead box O (FOXO) is a subfamily of the forkhead transcription factor family that plays an important role in cell fate decisions [73]. Lin et al. revealed a critical function for METTL3-mediated m6A modification in the hypoxic tumor microenvironment and identified FOXO3 as an important target of METTL3 in the resistance of HCC to sorafenib therapy [74]. The SOX family is associated with cell stemness [75]. In and glioma stem-like cells, METTL3 mediated SOX2 expression through an m6A-dependent mechanism to promote RNA stability and then increase stem cell frequency [76] (Fig. 2f). In addition, METTL3 could also promote SOX2 expression by elevating the level of AFF4, a transcriptional activator of SOX2, in an m6A dependent manner [77]. Notably, FOXO and SOX family members are involved in cancer stemness maintenance, embryonic cell development and somatic cell reprogramming [73, 75, 78]. Drugs directly targeting the FOXO and SOX families might impair normal development. A dose-dependent m6A methyltransferase-targeting strategy might cause a moderate alteration in FOXO and SOX expression. Future studies are needed to find a balance between cancer stemness inhibition and normal biological development.

Together, the current studies have shown the crucial roles of m6A methyltransferases in regulating key genes and pathways in cancer biology. Remarkably, in addition to certain forms of leukemia, in which tumorigenesis appears to be driven by a single oncogene, most cancers are the results of genetic alterations of a large number of genes that function through a relatively small number of pathways and processes [79]. Thus, the best hope for therapeutic development may lie in targeting altered pathways. The observations that m6A methyltransferases can broadly target nodal points and their upstream and downstream genes will likely contribute to the development of novel treatment strategies.

It is worth to note that, in addition to regulate protein coding genes, m6A methyltransferases also play a role on non-coding RNAs (ncRNAs) [2]. METTL3 and METTL14 mark m6A on pri-miRNA to facilitating miRNA processing [2, 3]. METTL3 could also regulate the RNA-RNA interaction, RNA–protein interaction, and chromatin remodeling function of lncRNAs in an m6A dependent manner [2]. In circRNAs, m6A methyltransferases could promote the biogenesis and protein synthesis of circRNAs or inhibit circRNA immunity. In some cases, lncRNAs can influence m6A methyltransferase in turn. For example, lncRNAs ARHGAP5-AS1 and LINC00470 have been revealed to guide METTL3 to specific targets to promote tumorigenesis in gastric cancer [19]. As lncRNAs were reported to be capable in modulating the catalytic activity of metabolic enzyme [80] and histone methyltransferase [81], it would be interesting to investigate whether it is also true for m6A methyltransferases. Importantly, m6A on ncRNAs is dynamically regulated by m6A methyltransferases during pathological processes [82], including tumorigenesis, providing a new direction for exploring the underlying molecular mechanism of m6A methyltransferases.”

## m6A-independent functions of m6A methyltransferases in cancer

In addition to the m6A writer complex that mediates m6A installation, m6A methyltransferases can function in an m6A-independent manner to regulate cellular processes. In human lung cancer, METTL3 has been reported to play an important role in promoting the translation of a subset of target mRNAs independent of its catalytic activity [83]. This study revealed the powerful function of METTL3 not only in enhancing the m6A modification pathway in the nucleus but also in facilitating oncogene translation in the cytoplasm, which regulates cancer cell growth, survival, and invasion. The multiple functions of METTL3 in cancer also indicate the need for exploiting specific METTL3 inhibitors that discriminate m6A-dependent and m6A-independent functions. In addition, other components in the MTC have been shown to have m6A-independent functions. For example, HAKAI has been reported to be an E3 ubiquitin-protein ligase that binds to E-cadherin, modulating epithelial cell–cell contacts and cell motility [84]. RBM15 and RBM15B act as cofactors of the nuclear receptor NXF1 to regulate mRNA export [85]. VIRMA can facilitate breast cancer by regulating CDK1 mRNA expression without changing the m6A modification in CDK1 [86]. Similarly, in gastric cancer cells, VIRMA promotes gastric cell progression mainly by directly binding to the 3’UTR of c-Jun mRNA to regulate c-Jun expression rather than altering m6A modification [87]. Notably, m6A methyltransferases can function in both an m6A-dependent and m6A-independent manner in a given cancer. Whether these two pathways engage in cross talk or have mutually exclusive functions remains unknown.

Overall, both the m6A-dependent and m6A-independent regulation of methyltransferases highlights their crucial roles in modulating the key signaling pathway of cancer, providing a wealth of new opportunities for treatment.

## The function of m6A methyltransferases in therapy resistance

Therapy resistance is the major cause of cancer treatment failure. Generally, resistance to cancer therapies can be classified as primary (intrinsic) resistance, manifesting by a lack of an objective clinical response following therapy, and secondary (acquired) resistance, which occurs after an initial tumor response [88, 89]. Therefore, exploring the mechanism by which cancer cells are resensitized to existing therapy and/or identifying novel druggable targets is of great clinical importance. Importantly, recent advances in the function of m6A methyltransferases have shown the potential in overcoming therapy resistance. We therefore focused the following discussion on the functions of m6A methyltransferases in chemotherapy, targeted therapy, immunotherapy and radiotherapy.

## m6A methyltransferases in chemotherapeutic resistance

**Fig. 3:**
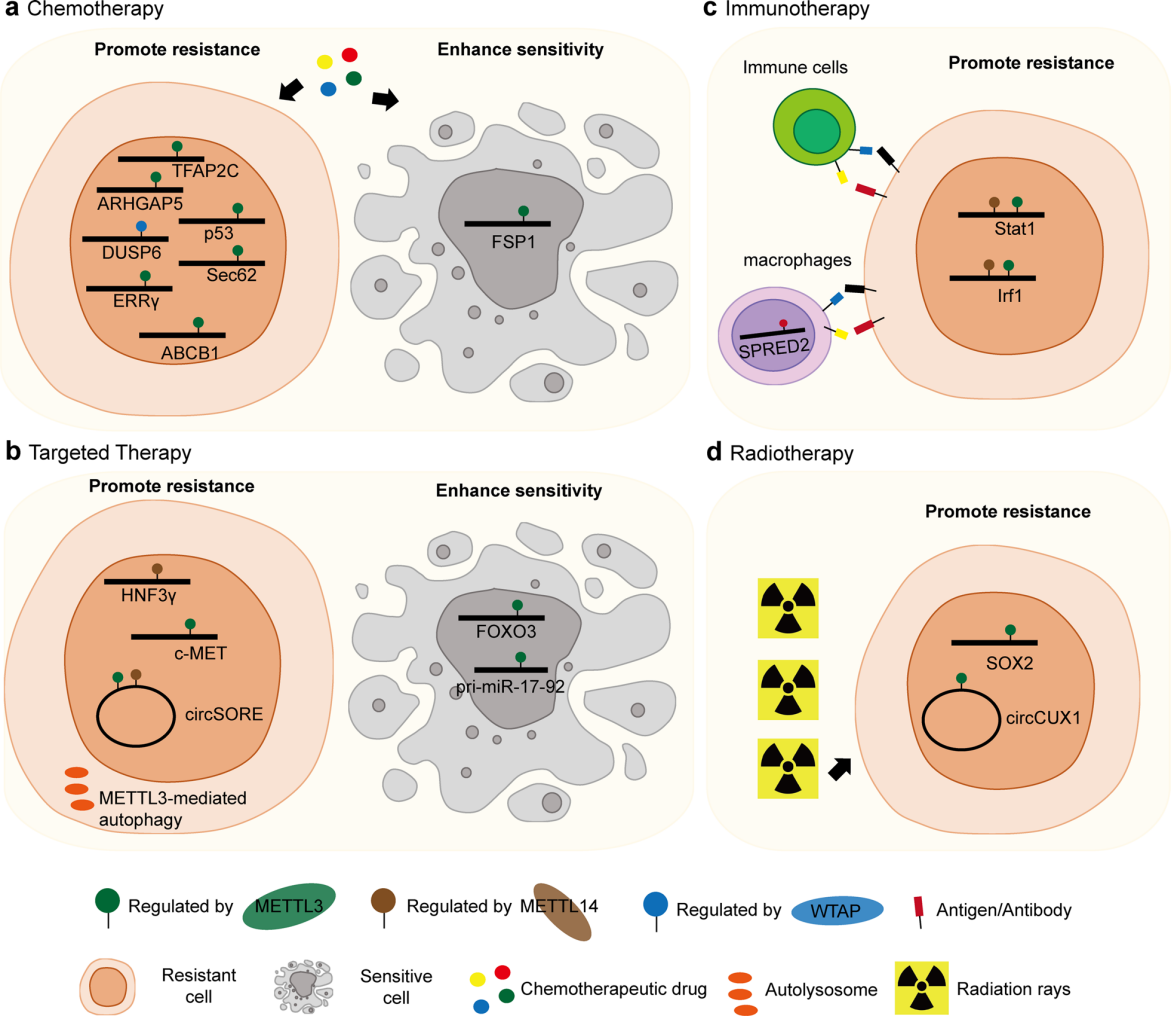
Overview of multiple functions of m6A methyltransferases in therapy resistance. As the key regulator of m6A, m6A methyltransferases have been found to regulate resistance to various therapy including chemotherapy (**a**), targeted therapy (**b**), immunotherapy (**c**) and radiotherapy (**d**)

Chemotherapy is among the most potent clinical strategies, and dramatic success has been achieved through the chemotherapeutic treatment of some malignant diseases [90]. However, resistance remains the major obstacle to effective chemotherapy. Excitingly, recent progress in RNA epigenetics has highlighted the functions of m6A methyltransferases in the resistance of a wide range of chemotherapeutic drug types, including platinum drugs, plant-based drugs, anthracycline antibiotics, and antimetabolites (Fig. 3a).

Platinum (Pt) drugs have been among the most widely used anticancer drugs for more than 40 years [91]. It has been reported that m6A methyltransferases contribute to the resistance to cisplatin, the first approved Pt drug. In testicular germ cell tumors (TGCTs), METTL3 mediates m6A on TFAP2C mRNA and promotes its stability by recruiting IGF2BP1, thus enhancing cisplatin resistance, probably by upregulating the expression of DNA repair-related genes [92]. In gastric cancer, METTL3, guided by an antisense lncRNA of ARHGAP5, ARHGAP5-AS1, methylates ARHGAP5 mRNA and promotes its stability to enhance cisplatin resistance [93]. Nonetheless, METTL3 may also have antitumor functions by enhancing chemotherapy sensitivity. In non-small cell lung carcinoma (NSCLC), METTL3 promotes the expression of FSP1, which enhances FSP1-mediated ferroptosis induced by cisplatin treatment [94]. In addition to METTL3, WTAP has also been associated with cisplatin resistance. In nasal-type natural killer/T-cell lymphoma (NKTCL), WTAP contributes to cancer progression and chemotherapy sensitivity by stabilizing DUSP6 mRNA in an m6A-dependent manner [95]. In addition to cisplatin, oxaliplatin is a novel platinum drug that is used to circumvent cisplatin-mediated resistance mechanisms. Two recent studies have shown that silencing METTL3 renders colorectal cancer cells more sensitive to oxaliplatin by targeting p53 and the Sec62-Wnt/β-catenin axis [62, 96]. Together, these findings indicate that targeting m6A methyltransferases might enhance the efficacy of Pt drugs and broaden their applicability to more tumor types.

Paclitaxel (Taxol), a plant-derived molecule, is now used as a drug against cancers that are generally considered to be refractory to conventional chemotherapy [97]. Chen et al. reported that METTL3 regulates the splicing of ERRγ induced by m6A installation, which promotes the expression of ERRγ to dictate Taxol resistance [98]. Notably, various plant-based drugs are well known for their anti-tubulin effect, similar to the function of Taxol, including vinca and colchicine [97]. It would be of clinical benefit to investigate whether m6A methyltransferases can confer chemotherapeutic sensitivity to these agents.

Although anthracycline chemotherapy regimens play prominent roles in several cancer treatments, they cause dose-related side effects due to induced cardiovascular toxicity [99]. A recent study showed that METTL3 promotes adriamycin resistance of MCF-7 breast cancer cells by accelerating pri-microRNA-221-3p maturation [100]. Additionally, targeting METTL3 can also cause anthracycline resistance through the downregulation of ABCB1 expression [98]. These studies indicate a potential strategy to lower cardiovascular side effects by modulating m6A methyltransferase activity, which might help to resensitize cancer cells to anthracycline and thus reduce the drug dose.

In addition, alkylating agents, which induce cytotoxic DNA damage as their main mode of action, constitute a class of frontline chemotherapeutic drugs [101]. Remarkably, METTL3 has been reported to play an important role in the DNA damage response: When METTL3 was silenced, cells showed delayed repair of DNA and elevated sensitivity to DNA damage [102, 103]. These observations indicate that targeting METTL3 may contribute to the sensitivity of alkylating agents by modulating DNA repair. Future studies are warranted to explore whether and how to leverage the DNA damage repair pathway to improve the clinical efficacy of alkylating agent-based cancer treatment.

Together, these studies suggest that, as writers of m6A modification, methyltransferases and their components can regulate chemotherapy resistance in a variety of cancers. Notably, targeting m6A methyltransferases can lead to attenuation of chemotherapy resistance to different types of drugs in a given cancer. In pancreatic cancer, METTL3-knockdown cells have been shown to exhibit higher sensitivity to a panel of anticancer reagents, including cisplatin, and two antimetabolites, gemcitabine and 5-Fu, as well as to irradiation [104]. Although this study did not identify the direct target genes of METTL3 action, a gene ontology analysis showed that METTL3 is associated with mitogen-activated protein kinase cascades, ubiquitin-dependent processes, RNA splicing and regulation of cellular processes, suggesting versatile roles for METTL3. These observations provide the rationale to combine an m6A methyltransferase-targeting strategy with various chemotherapy agents, which might be beneficial to a larger range of cancer types.

## m6A methyltransferases in targeted therapy

Targeted therapy developed to affect specific targets, such as enzymes or receptors, has been used in practice and shows great promise in modern cancer treatment. However, drug resistance is a formidable problem in the current era of molecularly targeted drugs [105–107].

Everolimus is a potent protein kinase inhibitor of the mTOR serine/threonine kinase signal transduction pathway. Gastric tumors with high METTL3 expression show preferred sensitivity to everolimus. Mechanistically, METTL3 mediates pri-miR-17-92 maturation in an m6A/DGCR8-dependent manner to suppress PTEN or TMEM127 and activate the AKT/mTOR pathway [53]. Crizotinib is an ATP-competitive inhibitor targeting ALK/ROS1/c-MET kinases, which are used as the first-line chemicals for the treatment of NSCLC with *ALK* mutations. However, the incidence of *ALK* mutations in NSCLC is relatively low, at only 3–7%, which severely limits the clinical application of crizotinib. Ding et al. showed, for the first time, that chidamide, a novel histone deacetylase inhibitor of the benzamide class of agents, can sensitize NSCLC cells to crizotinib by targeting c-MET. Intriguingly, the mechanism by which chidamide decreases c-MET expression is m6A-dependent; that is, in this study, chidamide reduced the stability and translational efficiency of METTL3 and WTAP and subsequently impaired the m6A level of c-MET [108]. Notably, c-MET overexpression is observed in 35–72% of NSCLC patients [109]. Thus, this study indicates that targeting m6A methyltransferases might largely expand the clinical application of crizotinib in NSCLC treatment. In addition to chidamide, β-elemene, the main active ingredient in the anticancer drug elemene, extracted from the Chinese medicinal plant Curcuma Wenyujin, has also been reported to be capable of inhibiting METTL3 through a different mechanism than that of the aforementioned chidamide. It has been speculated that β-elemene can directly bind and target the S-adenosylmethionine-binding domain of METTL3, showing the potential to inhibit METTL3 activity. Indeed, treatment with β-elemene can reverse the cell resistance to the drug gefitinib, an orally active and selective epidermal growth factor receptor (EGFR) inhibitor, by suppressing METTL3-mediated autophagy in NSCLC [110]. The discovery of chidamide and β-elemene attenuation of therapy resistance through their functions as potential inhibitors of m6A methyltransferases encourages further investigation to identify possible m6A modulators among existing drugs, including those that have not been reported to regulate methyltransferases. The crucial role of m6A methyltransferases in targeted therapy resistance is also supported by a recent study showing that METTL3 induces intrinsic resistance to gefitinib by positively regulating c-MET expression and activating the PI3K/AKT pathway in lung adenocarcinoma [111].

With regard to sorafenib, an oral multitargeted receptor tyrosine kinase inhibitor, the situation is complicated since m6A methyltransferases have been reported to play opposite roles in intrinsic and acquired sorafenib-resistant in HCC. METTL14-mediated HNF3γ reduction, which leads to downregulated OATP1B1 and OATP1B3 expression and thus reduces sorafenib uptake, causes HCC dedifferentiation and intrinsic sorafenib resistance [112]. Similarly, by establishing HCC sorafenib-resistant (SR) cell lines (HepG2-SR, SKhep1-SR, Huh7-SR and LM3-SR cells), Xu et al. found that METTL3 and METTL14 increase the stability of circRNA-SORE, which sequesters miR-103a-2-5p and miR-660-3p by acting as a microRNA sponge, thereby competitively activating the Wnt/β-catenin pathway and inducing sorafenib resistance [42]. These studies suggest oncogenic roles for METTL3 and METTL14 in sorafenib resistance by methylating protein-coding or noncoding genes. In contrast, METTL3 has been reported to methylate the 3’ untranslated region of FOXO3 mRNA to increase its stability, thereby restoring m6A-dependent sorafenib sensitivity in required sorafenib resistance of patients undergoing long-term sorafenib treatment [74]. This study identified a tumor suppressor of METTL3 in sorafenib resistance.

Overall, the important roles of m6A methyltransferases in intrinsic and acquired targeted drug resistance are gradually being recognized (Fig. 3b), which holds great promise for the translation of targeted therapeutic resistance in the future. However, we also need to emphasize that the context-dependent functions of m6A methyltransferases in cancer resistance, such as the opposite roles of METTL3 and METTL14 in sorafenib resistance, and related factors (e.g., genetic/epigenetic heterogeneities of cancer cell lines and primary tumor specimens) that affect the functions of m6A methyltransferase in a given targeted therapy need to be better understood.

## m6A methyltransferases and immunotherapy resistance

Immunotherapy, treatment of disease by inducing, enhancing or suppressing an immune response, has become an unprecedented treatment modality used against multiple cancers [89]. In particular, tumor-associated macrophages (TAMs) constitute a major component of the tumor microenvironment and are often associated with poor prognosis and immunotherapy resistance in patients [113]. Therefore, understanding the signaling involved in the activation of TAMs will help in developing better strategies for cancer immunotherapy. In an RNA-binding protein (RBP)-focused CRISPR-Cas9 screening performed to identify regulators of the innate response of macrophages, METTL3 was identified as a top candidate gene that regulates macrophage activation. In a mouse model, Mettl3 deficiency in macrophages resulted in an m6A-dependent reduction in *Irakm* mRNA and subsequently suppression of TLR signaling, attenuating the ability of macrophages to eliminate tumors in vivo [114]. This study indicates that METTL3-mediated m6A modification is required for the proper activation of macrophages. Similarly, Yin et al. [57] found that ablation of Mettl3 in macrophages reshapes the tumor environment by enhancing TAM infiltration into tumors, leading to increased tumor growth and metastasis in vivo. Mechanistically, loss of METTL3 expression impairs the YTHDF1-mediated translation of SPRED2, which enhances the activation of NF-κB and STAT3 through the ERK pathway. Importantly, Mettl3 depletion in macrophages impairs PD-1 blockade therapeutic efficacy in B16 melanoma, suggesting that METTL3 in macrophages may synergize with PD-1-based therapy [57]. Notably, macrophage-centered strategies, including macrophage targeting and TAM tumor-promoting blockades, have been entered into clinical trials and show great clinical potential [115, 116]. Thus, activating m6A methyltransferases might also show promise in macrophage-based immunotherapy.

In addition to their functions in immune cells, m6A methyltransferases in cancer cells can regulate the immune response. Wang et al. showed that loss of m6A modification through the depletion of the methyltransferases METTL3 and METTL14 enhanced the response to anti-PD-1 treatment in colorectal carcinoma and melanoma. This outcome is accomplished by promoting IFN-γ-Stat1-Irf1 signaling by stabilizing Stat1 and Irf1 mRNA when Mettl3 or Mettl14 is lost [117]. This study indicates that m6A methyltransferase inhibition may resensitize cancer cells to anti-PD-1 treatment.

Together, these studies revealed novel functions of m6A methyltransferases in the immune response and identified METTL3 and METTL14 as potential therapeutic targets in anticancer immunotherapy (Fig. 3c). However, these studies also raised concerns, indicating that we need to be very cautious when targeting METTL3 in melanoma. As described above, to eliminate melanoma, METTL3 expression needs to be activated in macrophages and inhibited in cancer cells. This observation indicates a need for a cell type-specific m6A methyltransferase-targeting strategy. In addition, the role of m6A methyltransferases in immunotherapy against a wide range of cancers still needs to be determined.

## m6A methyltransferases in radiotherapy resistance

Radiotherapy is used to kill cancer cells or prevent cancer growth by inducing DNA damage through high-energy rays (ionizing radiation). Compared to chemotherapy, radiotherapy has the advantage of local tumor control with relatively few systematic side effects. Approximately one-half of all cancer patients receive radiotherapy as part of their treatment [56, 118]. However, the acquisition of resistance limits the efficacy of radiotherapy. As a versatile modification, m6A and related methyltransferases also regulate radiotherapy resistance (Fig. 3d). In GBMs, silencing METTL3 in GSCs has been shown to reduce DNA repair and enhance sensitivity to γ-irradiation. Mechanistically, METTL3 methylates the 3’UTR of SOX2, which enhances its stability and promotes GSC maintenance [77]. In hypopharyngeal squamous cell carcinoma (HPSCC), malignant tumors with one of the worse prognoses, METTL3 confers radioresistance by promoting the stability of circCUX1. Interestingly, circCUX1 binds to Caspase1 mRNA and inhibits its expression, resulting in a decrease in programmed cell death, thereby enabling tolerance to radiotherapy [119].

Together, these studies reveal the essential roles of METTL3 in radioresistance, which may open new possibilities in radiotherapy and translational medicine. In particular, in addition to its ability to mediate DNA damage-induced cancer cell death, radiotherapy can have profound immunostimulatory effects [56] and, therefore, is increasingly viewed as a promising combination partner of immuno-oncology agents. Thus, given the crucial roles of m6A methyltransferases in the immune response, it will be interesting to investigate their functions in radiotherapy in the tumor microenvironment, especially in an environment involving immune cells.

Overall, these findings highlight the importance of m6A methyltransferases as key regulators in gene expression and disease states. The crucial roles of m6A methyltransferases in resistance to various cancer therapies (summarized in Fig. 3 and Table 1) encourage the development of m6A methyltransferase-targeting strategies and the investigation of their potential application in the clinic.

**Table 1:** The functions of m6A methyltransferases in drug and radiotherapy resistance

Therapy type	Drug name	Cancer	Regulator	Regulation	Target	References
Chemo-therapy	Cisplatin	Seminoma	METTL3	PR	TFAP2C	[92]
		Gastric cancer	METTL3	PR	ARHGAP5	[93]
		NSCLC	METTL3	ES	FSP1	[94]
		NKTCL	WTAP	PR	DUSP6	[95]
		Nasopharyngeal carcinoma	METTL3	PR	TRIM11	[43]
	Oxaliplatin	Colorectal cancer	METTL3	PR	P53/Sec62	[96]
	Taxol	Breast cancer	METTL3	PR	ERRγ	[98]
	Doxorubicin (adriamycin)	Breast cancer	METTL3	PR	pri-microRNA-221-3p	[100]
	Anthracycline	Breast cancer	METTL3	PR	ABCB1	[98]
Targeted therapy	Everolimus	Gastric tumors	METTL3	ES	pri-miR-17–92	[53]
	Crizotinib	NSCLC	METTL3/WTAP	PR	c-MET	[108]
	Gefitinib	NSCLC	METTL3	PR	METTL3-mediated Autophagy	[110]
		Lung Adenocarcinoma	METTL3	PR	c-MET	[111]
	Sorafenib	HCC	METTL14	PR	HNF3γ	[112]
		HCC	METTL3/14	PR	circRNA-SORE	[42]
		HCC	METTL3	ES	FOXO3	[74]
Immuno therapy	Anti-PD-1	Melanoma	METTL3 (macrophages)	PR	SPRED2	[57]
		Colorectal carcinoma/Melanoma	METTL3/14	PR	Stat1/Irf1	[117]
Radio-therapy	γ-irradiation	Glioblastoma	METTL3	PR	SOX2	[76]
	X-ray Irradiation	Hypopharyngeal Squamous cell carcinoma	METTL3	PR	circCUX1	[119]

*NSCLC* non-small cell lung carcinoma, *NKTCL* nasal-type natural killer/T-cell lymphoma, *HCC* hepatocellular carcinoma, *PR* Promote resistance, *ES* Enhance sensitivity

## Potential clinical application of targeting m6A methyltransferases

There is growing interest in developing m6A writer-based strategies to combat cancer. Pharmacological modulation targeting epigenetic pathways by small-molecule modulators holds immense therapeutic promise for advancing traditional medicine. Remarkably, METTL3 has several properties that make it an ideal target: (1) It is an enzyme critical for m6A modification of most mRNAs; (2) it is dynamically regulated; and (3) it contains an SAM-binding pocket that has been successfully targeted in other methyltransferases [120]. Since METTL3 has opposing, context-dependent roles in cancer, interventions to both stimulate and inhibit the activity of METTL3 can be proposed as cancer therapies. In addition to chemicals targeting METTL3, recent research progress has shown the potential of limiting SAM levels, ncRNAs and programmable m6A-editing systems in m6A methyltransferase-based cancer treatment. METTL3 chemical modulators are listed in Table 2.

**Table 2:** List of METTL3 modulators

Type	Function	Name/Company	IC_50_/EC_50_	Cancer	Estimated phase I trial start date	References
Activator	Specific activator	Compound 3	117 nM	ND	ND	[121]
Inhibitor	Pan-MTase inhibitor	Sinefungin	2.36μΜ	ND	ND	[121]
		3-deazaadenosine	ND	ND	ND	[129]
	Specific inhibitor	Compound 2	8.7 μM	ND	ND	[126]
		UZH1a	280 nM	AML	ND	[127]
		STM2457/STORM Therapeutics	16.9 nM	AML	2021	[128]
		Accent Therapeutics	ND	AML, NSCLC	2021, 2022	[120]

*AML* acute myeloid leukemia, *NSCLC* non-small cell lung cancer, *ND* no data available

## Activators of m6A methyltransferases

The METTL3-METTL14 complex, a SAM-dependent methyltransferase (MTase), catalyzes the transfer of a methyl group from SAM to adenine in single-stranded RNA [22, 23]. Thus, the ability to bind SAM is crucial for METTL3-METTL14 complex activity. A recent study identified the binding site of SAM as a target area for potential METTL3-METTL14 ligands and identified four compounds as activators of the METTL3-METTL14-WTAP (METTL3-14-WATP) complex by virtual screening of molecular libraries [121]. The binding mode of the compounds overlapped with the METTL3-14-WATP active site, which allows the close proximity of the compound interacting with SAM. This close interaction may increase the binding affinity of METTL3 for SAM and lower the energy barrier of the substrate RNA methylation reaction, thereby contributing to enhanced METTL3-14-WATP activity. Upon validation of the functions of these compounds in HEK293 cells, compounds 1–3 were shown to increase the relative m6A amount, suggesting that activation of METTL3-14-WATP, as observed in silico and in vitro experiments, is biologically translatable at the cellular level. Importantly, compound 3, which has been shown to increase mRNA m6A by more than 40% at 1 nM, may act as the lead compound for further development of more-potent activators of the METTL3-enzyme complex [121]. Overall, this study discovered the first m6A methyltransferase activators. However, several issues remain to be addressed. First, although no cytotoxicity was observed at concentrations up to 100 mΜ for all the compounds tested with HEK293 cells, further cytotoxicity analyses with a larger panel of cell lines and a mouse model are still needed. Second, further investigations to evaluate the selectivity of these compounds for METTL3-14-WATP against other RNA, DNA and protein methyltransferases are warranted. Third, the anticancer potential of these compounds needs to be studied in renal cell carcinoma, glioblastoma and bladder cancer, where METTL3 plays a tumor suppressor role.

## Inhibitors of m6A methyltransferases

Given the critical role of SAM binding in methylation activity, many analogs of SAM or SAH, a product of SAM-dependent transmethylation reactions, have been synthesized as SAM competitors to inhibit the function of methyltransferases [122]. Sinefungin, a natural nucleoside analog of SAH, is a pan-MTase inhibitor [123]. Interestingly, sinefungin inhibits the activity of the SARS-CoV-2 nsp16 MTase, indicating the potential application of sinefungin as an antiviral agent [124]. In addition, a recent study showed that sinefungin expectedly inhibited METTL3-14-WATP activity with an IC_50_ of 2.36 μΜ [121]. This observation indicates that pan-MTase inhibitors can also function as m6A inhibitors. In addition to sinefungin, methylthioadenosine is one of the most popular pan-inhibitors of MTase, as it is cell permeable [125]. The role of methylthioadenosine in modulating m6A methyltransferases requires further evaluation. Notably, the observed IC_50_ of sinefungin correspond to the IC_50_ values of other methyltransferases, raising the need to develop more-sensitive and more-potent inhibitors of m6A methyltransferases. In addition, specific m6A methyltransferase inhibitors may reduce the side effects caused by pan-MTase inhibitors because they influence the functions of DNA and protein methyltransferases.

**Fig. 4:**
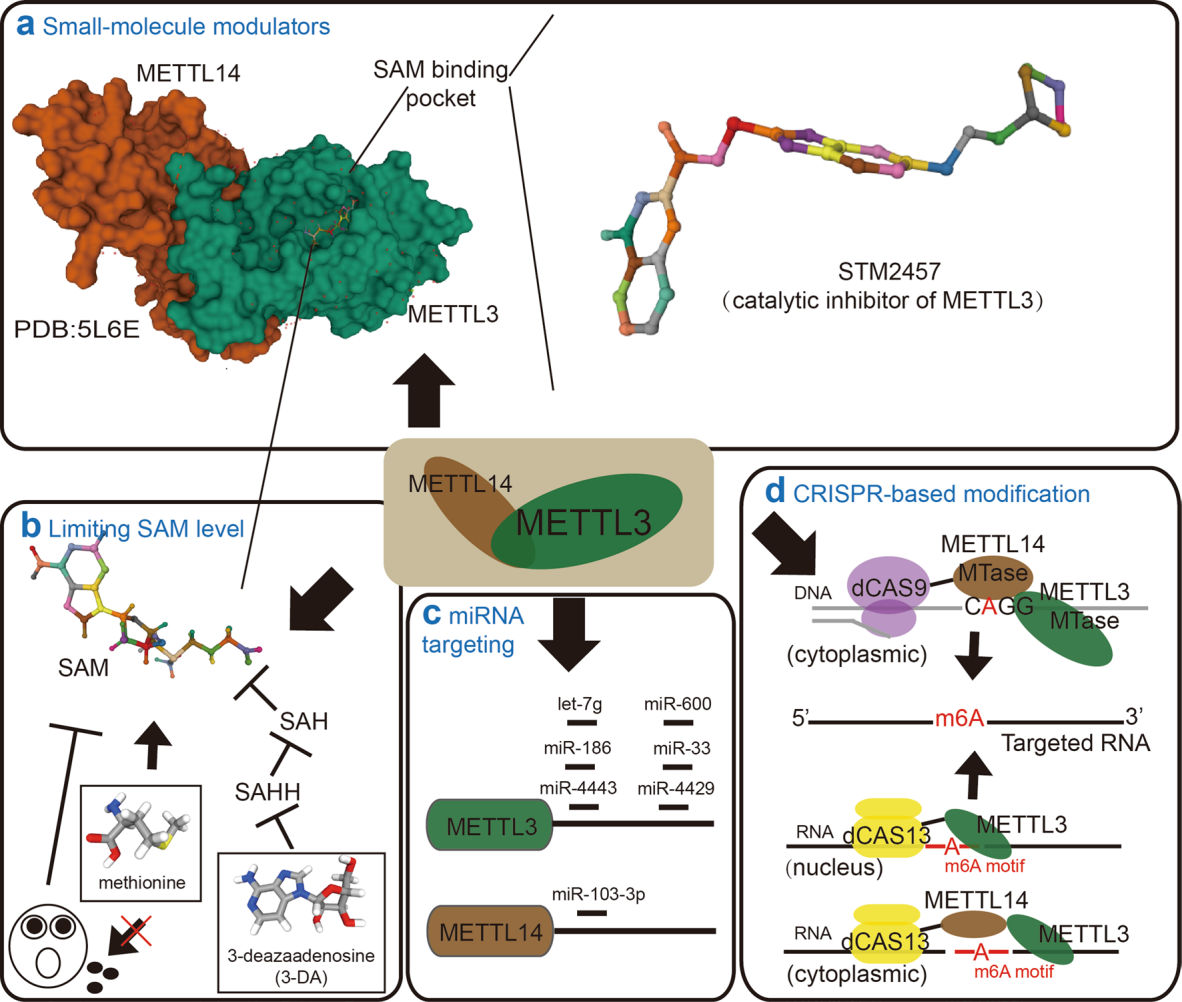
Potential approaches for targeting m6A methyltransferases. **a** Targeting strategy with small-molecule modulators. Schematic domain structure of the METTL3-METTL14 complex with SAM binding (left). STM2457, a catalytic inhibitor of METTL3 that competes with SAM for the SAM-binding pocket, represents the general strategy of m6A methyltransferase modulator development (right). **b** Targeting strategy related to limit SAM level. Restriction of SAM levels by 3-deazaadenosine (3-DA) reduces m6A levels by blocking SAHH and subsequently elevating SAH levels. Specifically, limiting the level of dietary methionine, which is required for SAM synthesis, can also regulate METTL3 activity. **c** Targeting strategy for the use of specific miRNAs to inhibit METTL3/METTL14 expression. **d** Targeting strategy of CRISPR-based site-specific m6A modification

In an effort to discover specific inhibitors of METTL3, a recent study screened a library of 4000 analogs and derivatives of SAM by high-throughput docking on METTL3, identifying 70 compounds for experimental validation. Among these candidates, one compound (compound 2) showed good ligand efficiency and the most favorable inhibitory potency, with an IC_50_ of 8.7 μM [126]. However, the lack of cell activity data hinders the evaluation of the true effect of this compound on METTL3 and m6A. Since METTL3 has been linked to the initiation and maintenance of AML, two other studies have examined the cellular activity of a new METTL3 inhibitor for use in treating AML. One study has identified a METTL3 inhibitor (UZH1a) that showed potency at a 280 nM concentration in a biochemical assay [127]. UZH1a fills the pocket of the adenosine moiety of SAM but not the pocket of the SAM methionine, which makes it more selective of METTL3 than other SAM-dependent MTases. The effect of METTL3 inhibition by UZH1a directly translates into m6A level depletion and impaired growth of MOLM13 leukemia cells [127]. Another group reported a more exciting study showing the first-in-class catalytic inhibitor of METTL3 (STM2457) (shown in Fig. 4a) with an IC_50_ of 16.9 nM [128]. STM2457 is highly specific for METTL3 and shows no inhibition of other RNA methyltransferases or kinases, which may be due to its structural dissimilarity with SAM or any known methyltransferase inhibitor and its avoidance of the homocysteine binding pocket that is utilized by SAM. Treatment with STM2457 leads to reduced cell proliferation rates and an increase in differentiation and apoptosis rates in various leukemia cell lines. Furthermore, oral doses of STM2457 impaired engraftment and prolonged survival in AML mouse models, as well as a patient-derived xenograft model. Importantly, STM2457 had no effect on normal hematopoietic stem and progenitor cells, peripheral blood counts or mouse body weight [128]. These results highlight that pharmacological inhibition of METTL3 by STM2457 is a potential therapeutic strategy against AML.

STM2457 is produced by STORM Therapeutics company, and STORM is now investigating the application of STM2457 in the treatment of other tumor types, including solid tumors, and is planning to conduct a phase I trial in 2021 [120]. Furthermore, two other lead companies in this space are working on METTL3 inhibitors. Accent Therapeutics has discovered METTL3 inhibitors and plans to conduct phase I trials in 2021 for patients with AML and NSCLC. Gotham Therapeutics, with a METTL3 inhibitor in preclinical development, is aiming for a clinical trial in 2022 for patients with AML [120]. As companies start developing inhibitors of m6A methyltransferases with proof-of-concept studies, RNA epigenetic drug development is entering a new era.

## Modulation of SAM/SAH levels to influence the function of m6A methyltransferases

In addition to SAM-competitive inhibitors, restriction of SAM levels may also lead to inhibited m6A methyltransferase activity and reduced global m6A levels (Fig. 4b). A recent study has shown the promise of targeting m6A methyltransferase by a drug modulating SAM metabolism. It has been suggested that METTL14 plays an oncogenic role in breast cancer. Treatment with 3-deazaadenosine (3-DA), a general methylation inhibitor that depletes SAM by blocking S-adenosylhomocysteine hydrolase (SAHH) activity, reduces m6A levels and dramatically suppresses the migration and invasion of cancer cells [129]. This study indicated that targeting the synthetic pathway of SAM with chemical inhibitors can modulate the activity of m6A methyltransferases. Notably, SAM can be converted into methionine, an essential amino acid that cannot be synthesized de novo in humans [130, 131]. Thus, the levels of methionine obtained from the diet can have a profound impact on cellular methionine metabolism, which establishes a link between nutrition and tumor cell metabolism that may allow for regulating m6A methyltransferase activities through diet. The feasibility of this strategy has been shown with a preliminary study on autosomal dominant polycystic kidney disease (ADPKD), the most common monogenetic human disorder. In an ADPKD mouse model, SAM supplementation aggravated cyst growth by upregulating Mettl3 expression. In contrast, a methionine-restricted diet reduced kidney size and cysts compared with a standard diet by inducing the expression of Mettl3 [132]. Although the underlying mechanism by which SAM and methionine regulate Mettl3 expression is unclear, this study links methionine utilization to epitranscriptome activation of disease progression. Notably, limiting amino acids has been shown to have great application in the treatment of cancer [133]. In acute lymphoblastic leukemia (ALL), the combination of L-asparaginase, which limits asparagine level, with other chemotherapeutic regimens has led to overall survival rates of approximately 90% for ALL pediatric patients [134]. Given the crucial roles of m6A methyltransferases in cancer development and therapy resistance, it would be of clinical benefit to investigate the potential of targeting SAM metabolism and dietary methionine restriction as novel therapeutic interventions in cancer treatment.

## Targeting m6A methyltransferase by ncRNA

Consistent with the observations that alterations in m6A levels contribute to tumorigenesis, widespread dysregulation of m6A methyltransferases has been revealed in various cancer types [135, 136]. These results raise the possibility of targeting m6A methyltransferase through their regulatory machinery. miRNAs constitute a class of noncoding RNA molecules with powerful gene regulation potential. They bind to complementary target mRNAs, resulting in mRNA translational inhibition or degradation [137]. Notably, seven miRNAs have been found that directly target m6A methyltransferases in diseases (Fig. 4c). In breast cancer, miRNA let-7 g downregulates the expression of METTL3 by targeting its 3’UTR. Interestingly, METTL3 elevates the expression of HBXIP, which can inhibit let-7 g. The resulting positive feedback loop of HBXIP/let-7 g/METTL3/HBXIP leads to accelerated cell proliferation and inhibited apoptosis [138]. In gastric cancer, miR-4429 acts as a tumor suppressor by targeting METTL3 to inhibit m6A-induced stabilization of SEC62 and reduce tumor growth [139]. In addition, miR-186 [140] and miR-600 [141] have also been reported to target METTL3 and suppress hepatoblastoma and lung cancer progression, respectively. When targeting METTL3 in NSCLC, miRNAs can be tumor suppressor genes, as we illustrate above, or oncogenes due to the context-dependent roles of METTL3 in cancer progression. It has been reported that miR-33a is capable of reducing cell proliferation [142], while exosomal miR-4443 promotes cisplatin resistance in NSCLC [94]. Sun et al. found that, in addition to targeting METTL3, miR-103-3p can inhibit METTL14 expression and impair osteoblastic bone formation, which might result in osteoporosis [143]. These studies showed the powerful effects of miRNAs in regulating m6A methyltransferase expression. Remarkably, some miRNA mimics and molecules targeted to miRNAs (anti-miRs) have reached the stage of clinical development, including a mimic of the tumor suppressor miR-34, which was entered in phase I clinical trials for treating cancer, and anti-miRs targeted to miR-122, which was entered in phase II trials for treating hepatitis [144]. These advances have shown promise of miRNA-based therapeutics in targeting m6A methyltransferases. However, the observations that a particular miRNA can target multiple genes and that a given gene can be targeted by various miRNAs complicate the development of selective miRNA‑directed therapeutics. Thus, to identify the most efficacious therapeutic candidates, a clear picture of the miRNA targetome, with a definitive number of oncogenes or tumor suppressors targeted by a particular miRNA, is needed.

In addition to miRNAs, small interfering RNAs (siRNAs), antisense oligonucleotides (ASOs) and locked nucleic acids (LNAs) are promising therapeutic platforms useful for blocking the synthesis of disease-causing proteins [144], providing attractive alternative approaches to target m6A methyltransferases by ncRNAs.

## CRISPR-based site-specific m6A modifications

The critical roles of m6A methyltransferases have been widely studied. However, these studies have sometimes led to controversial observations. For instance, MALAT1-m6A modifications generated by the METTL3-METTL14 complex are important to the metastatic ability of esophageal cancer cells (ESCCs), but silencing METTL3 cannot recapitulate the phenotypes of impaired migration observed in MALAT1-Δm6A cells. This discrepancy might be the result of knocked down METTL3 expression affecting the m6A status of all the transcripts that integrate contributions from both oncogenes and tumor suppressor genes, not only MALAT1 [145]. These results highlight that (1) modulating the m6A on entire epitranscriptome or on a specific transcript causes different phenotypes and (2) disturbing the m6A modification of a single transcript is sufficient to attenuate cancer progression. Therefore, the development of a site-specific m6A installation system will not only help us to elucidate the function of regional methylation but also provide a powerful tool to correct dysregulated m6A modification on specific genes for cancer treatment.

Recent advances in CRISPR-based technologies have revolutionized biomedical research by enabling precision genome editing [146–148]. Two groups have recently developed a programmable m6A modification with a CRISPR-directed methyltransferase (Fig. 4d). Since the METTL3-METTL14 heterodimer possesses strong catalytic activity, they constructed a single-chain m6A MTase by linking two MTase domains derived from human METTL3 and METTL14 (referred to as M3M14) and fused it to dCas9 [149] and Cas13 [150], respectively. Although M3M14-dCas9 and dCas13-M3M14nes can direct site-specific m6A modification, they are predominately enriched in the cytoplasm, which is different than the localization of endogenous METTL3-METTL14, which cotranscriptionally methylates nascent mRNA within nuclear speckles. The cytoplasmic m6A by M3M14-dCas9 and dCas13-M3M14nes impedes the recognition of m6A-modified transcripts by nuclear m6A readers, which might lead to abnormal regulation of RNA fates through splicing, microRNA maturation, nuclear export and chromatin accessibility. Thus, a nucleus-localized targeted RNA methylation system is needed. When fusing a truncated METTL3 MTase domain to a nucleus-localized dCas13, the resulting dCas13-M3nls can deposit m6A on target mRNAs and enable m6A-dependent nuclear processing events [150]. A comparison of the three produced m6A writer systems showed that they all exhibit similar on-efficiency, but the lack of METTL14 RNA-binding domain within dCas13-M3nls greatly reduced off-target activity [150]. Additionally, dCas13-M3nls do not require any laboratory-synthesized modified PAMmer oligonucleotides used by M3M14-dCas9. Therefore, dCas13-M3nls are compatible with delivery strategies, including viral transduction, that cannot easily deliver cargo containing synthetic components. Notably, CRISPR has been widely applied to gene therapy in the scientific community, providing a powerful tool in precision medicine for cancer treatment, and several relevant clinical trials are currently in phase I/II [151]. The CRISPR-based m6A targeting system, offering site-specific methylation of specific oncogenes or tumor suppressor genes, not only facilitates mechanistic understanding of the epitranscriptome but also provides a potent approach to m6A-based precision medicine. Further studies are needed to optimize the efficiency and specificity of this approach.

## Conclusion and perspectives

Ever-increasing knowledge on m6A methyltransferases is revealing the overarching importance of these enzymes in tumorigenesis. However, current studies are mainly focused on the function of the METTL3-METTL14-WTAP complex in cancer, and other m6A methyltransferases may contribute to cancer initiation and maintenance by regulating RNA species- and site-specific m6A. Two studies have begun to show the oncogenic role of METTL5, an rRNA m6A methyltransferase, in breast cancer [152] and lung adenocarcinomas [153]. The function of the other rRNA m6A methyltransferase, ZCCHC4, in cancer is largely unknown. In addition, METTL16 has recently been reported to mediate 3' splice site m6A modification, which prevents the essential splicing factor U2AF35 from recognizing the 3' splice site, thereby inhibiting RNA splicing [154]. Importantly, splicing patterns are frequently altered in cancer, resulting in a growing interest in targeting splicing catalysts and splicing regulatory proteins in cancer treatment [155]. Thus, it will be important to investigate the function of METTL16 in the cancer context.

One of the most exciting discoveries related to the function of m6A in cancer biology is the targeting of m6A methyltransferase to rewire the cancer epitranscriptome to resensitize resistant cells to existing therapies. Therefore, a rational combination of small molecules that target m6A methyltransferases with chemotherapy, targeted therapy, immunotherapy, and/or radiotherapy is a promising therapeutic avenue to overcome therapy resistance. Reports of small molecules that target METTL3 in early preclinical studies have provided encouraging preliminary data. However, issues with potency, selectivity, and cytotoxicity may limit their clinical utility. In addition, the CRISPR-based m6A targeting system, which enables site-specific methylation, shows promise for use in precise medicine. Moreover, care needs to be taken to understand the underlying contexts in which m6A methyltransferases play oncogenic or tumor suppressor roles [156–158]. We are thankful for the modern clinical trial design, which allows the collection of samples from tumors and blood both before and after treatment. This paradigm may aid with the development of pharmacodynamic biomarkers of m6A methyltransferase inhibitory or activation effects in cancer patients.

The current interventions using m6A methyltransferase inhibitors or activators are largely based on the catalytic activity of the enzyme. However, the independent catalytic functions of these methyltransferases also contribute to cancer biology. In lung cancer, METTL3 regulates the m6A modification and stability of JUNB mRNA to facilitate the metastasis of cancer cells [159]. On the other hand, METTL3 promotes the translation of important oncogenes by interacting with eIF3h to circularize mRNA [160]. Notably, in contrast to wild-type METTL3, forced expression of either a catalytically inactive mutant or METTL3 (A155), which disrupts the interaction of METTL3 with translation initiation factors eIF3h, is unable to promote the invasive capability of lung fibroblasts [160]. These observations strongly indicate that both the catalytic-dependent and catalytic-independent functions of METTL3 are crucial for its oncogenic role in cancer. Thus, future efforts aimed at inhibiting the METTL3-eIF3h association may lead to the development of new cancer therapies. Notably, Proteolysis targeting chimeras (PROTACs), which are heterobifunctional small molecules that degrade proteins of interest through ubiquitin proteasome system, are another promising technology for the development of m6A methyltransferase-targeting therapeutics [161]. In addition, although this review focuses mostly on m6A methyltransferases, studies of m6A erasers have identified several potent inhibitors targeting the demethylase FTO in acute myeloid leukemia [162, 163]. Additionally, in future investigations, m6A reader proteins, as well as other RNA modification regulators, should be taken into account as potential therapeutic targets in cancer treatment [164–166].

Altogether, we summarize the recent advances in understanding the crucial roles of m6A methyltransferases in regulating important cancer pathways and therapy resistance. Better and earlier identification of the exact function of m6A methyltransferases can provide key targets for drug development. This increased body of knowledge, coupled with comprehensive preclinical analysis, may enable m6A methyltransferase-targeting therapeutics to become clinical realities.

## Data Availability

The material supporting the conclusion of this review has been included within the article.

## Abbreviations

m6A: N6-methyladenosine
METTL3: Methyltransferase-like 3
METTL14: Methyltransferase-like 14
WTAP: Wilms’ tumour-associated protein
lncRNAs: Long noncoding RNAs
VIRMA(KIAA1429): Vir Like M6A methyltransferase associated
ZC3H13: Zinc finger CCCH-type containing 13
RBM15/15B: RNA binding motif protein 15/15B
HAKAI: Cbl proto-oncogene like 1
METTL5: Methyltransferase like 5
TRM112: TRNA methyltransferase subunit 11-2
ZCCHC4: Zinc finger CCHC-type containing 4
METTL16: Methyltransferase Like 16
miRNAs: MicroRNAs
snRNAs: Small nuclear RNAs
snoRNAs: Small nucleolar RNAs
rRNAs: Ribosomal RNAs
MeRIP-Seq: Methylated RNA immunoprecipitation sequencing
ALKBH5: AlkB Homolog 5
FTO: Fat mass and obesity associated
MTC: M6A methyltransferase complex
SAM: S-adenosylmethionine
5-Fu: 5-Fluorouracil
SARS-CoV-2: Severe acute respiratory syndrome coronavirus 2
CRISPR: Clustered regularly interspaced short palindromic repeats
AML: Acute myeloid leukemia
PC: Prostate carcinoma
OSCC: Oral squamous cell carcinoma
ccRCC: Clear cell renal cell carcinoma
HB: Hepatoblastoma
HCC: Hepatocellular carcinoma
CML: Chronic myelocytic leukemia
NPC: Nasopharyngeal carcinoma
SAH: S-adenosylhomocysteine
CRC: Colorectal cancer
RB: Retinoblastoma
MDR: Multidrug resistance
GBM: Glioblastoma
GSCs: Glioma stem cells
FOXO: Forkhead box O
EMT: Epithelial-mesenchymal transition
Pt: Platinum
TGCTs: Testicular germ cell tumors
NSCLC: Non-small cell lung carcinoma
NKTCL: Nasal-type natural killer/T-cell lymphoma
EGFR: Epidermal growth factor receptor
SR: Sorafenib-resistant
TAMs: Tumor-associated macrophages
RBP: RNA-binding protein
HPSCC: Hypopharyngeal squamous cell carcinoma
MTase: Methyltransferase
3-DA: 3-Deazaadenosine
SAHH: S-adenosylhomocysteine hydrolase
ADPKD: Autosomal dominant polycystic kidney disease
ALL: Acute lymphoblastic leukemia
siRNAs: Small interfering RNAs
ASOs: Antisense oligonucleotides
LNAs: Locked nucleic acids
ESCCs: Esophageal cancer cells
